# Supplementary material for: Content Analysis of Official Public Health Communications in Ontario, Canada during the COVID-19 Pandemic
Source: Int J Environ Res Public Health. 2024 Mar 15;21(3):351. doi: 10.3390/ijerph21030351 (PMC10970262; doi:10.3390/ijerph21030351)
Supplement: Supplementary file 1 [file ijerph-21-00351-s001.zip › MF_IJERPH_Codebook.pdf]

Ontario Public Health Releases

Codes

Name	Description	Files	References
Dates		322	645
April 2020		30	30
April 2021		16	16
April 2022		1	1
August 2020		7	7
August 2021		3	3
August 2022		2	2
December 2020		14	14
December 2021		8	8
December 2022		2	2
February 2020		6	6
February 2021		9	9
February 2022		3	3
January 2020		2	2
January 2021		11	11
January 2022		3	3
July 2020		13	13
July 2021		3	3
July 2022		2	2

Name	Description	Files	References
June 2020		26	26
June 2021		12	12
March 2020		32	32
March 2021		20	20
March 2022		1	1
May 2020		22	22
May 2021		12	12
November 2020		15	15
November 2021		5	5
October 2020		10	10
October 2021		7	7
October 2022		1	1
September 2020		17	17
September 2021		5	5
September 2022		2	2
Week 10, 2020		6	6
Week 10, 2021		2	2
Week 11, 2020		4	4
Week 11, 2021		4	4
Week 11, 2022		1	1
Week 12, 2020		10	10
Week 12, 2021		4	4
Week 13, 2020		8	8
Week 13, 2021		4	4
Week 14, 2020		8	8

Name	Description	Files	References
Week 14, 2021		7	7
Week 15, 2020		5	5
Week 15, 2021		3	3
Week 16, 2020		8	8
Week 16, 2021		3	3
Week 16, 2022		1	1
Week 17, 2020		9	9
Week 17, 2021		2	2
Week 18, 2020		6	6
Week 18, 2021		5	5
Week 19, 2020		5	5
Week 19, 2021		1	1
Week 2, 2021		2	2
Week 2, 2022		1	1
Week 20, 2020		3	3
Week 20, 2021		5	5
Week 21, 2020		4	4
Week 21, 2021		4	4
Week 22, 2020		7	7
Week 22, 2021		2	2
Week 23, 2020		5	5
Week 23, 2021		5	5
Week 24, 2020		8	8
Week 24, 2021		4	4
Week 25, 2020		7	7

Name	Description	Files	References
Week 25, 2021		3	3
Week 26, 2020		6	6
Week 26, 2021		3	3
Week 28, 2020		2	2
Week 28, 2021		1	1
Week 29, 2020		4	4
Week 29, 2022		1	1
Week 3, 2021		3	3
Week 3, 2022		1	1
Week 30, 2020		3	3
Week 30, 2022		1	1
Week 31, 2020		4	4
Week 31, 2021		2	2
Week 32, 2020		3	3
Week 33, 2020		2	2
Week 34 2022		1	1
Week 34, 2020		1	1
Week 34, 2021		1	1
Week 35, 2020		1	1
Week 35, 2021		1	1
Week 36, 2020		1	1
Week 36, 2021		1	1
Week 36, 2022		1	1
Week 37, 2020		3	3
Week 38, 2020		5	5

Name	Description	Files	References
Week 38, 2021		2	2
Week 39, 2020		5	5
Week 39, 2021		3	3
Week 39, 2022		1	1
Week 4, 2020		1	1
Week 4, 2021		3	3
Week 4, 2022		1	1
Week 40, 2020		7	7
Week 40, 2022		1	1
Week 41, 2020		2	2
Week 41, 2021		1	1
Week 42, 2020		2	2
Week 42, 2021		1	1
Week 42, 2022		1	1
Week 43, 2021		2	2
Week 44, 2020		1	1
Week 44, 2021		3	3
Week 45, 2020		5	5
Week 45, 2021		2	2
Week 46, 2020		3	3
Week 46, 2021		1	1
Week 47, 2020		4	4
Week 47, 2021		1	1
Week 48, 2020		5	5
Week 48, 2021		1	1

Name	Description	Files	References
Week 49, 2020		3	3
Week 49, 2021		2	2
Week 5, 2020		1	1
Week 5, 2021		2	2
Week 50 2022		1	1
Week 50, 2020		4	4
Week 50, 2021		2	2
Week 51, 2020		4	4
Week 51, 2021		2	2
Week 51, 2022		1	1
Week 52, 2021		2	2
Week 53, 2020		3	3
Week 6, 2021		2	2
Week 7, 2021		4	4
Week 7, 2022		2	2
Week 8, 2021		2	2
Week 8, 2022		1	1
Week 9, 2020		6	6
Week 9, 2021		1	1
Decisions are evidence-based, tapping both social and natural sciences		51	87
Risk management and risk communication processes are transparent	- Assumptions values, methods, and plans must be clear and accessible - When facts are unknown or uncertain, Health Canada will be clear about what gaps remain and what efforts are being taken to fill them - Must be clear about mistakes made and rectify them - When information has to be kept secret, need an explanation as to why	321	1676
Action Plan or Description		312	1090

Name	Description	Files	References
Address uniqueness		3	3
Case Descriptions		17	31
Explanation or Convincing the Public		33	45
Vaccines		70	102
Variants		22	28
Stakeholders are the focal point		270	1041
Businesses		29	57
Education		17	60
Elderly (help the eldery, appeal to the elderly)		36	78
Family		3	6
Healthcare and Frontline Workers		85	160
Other Workers		9	17
Public-centered		206	409
Researchers		1	4
Strategic risk communications is integral to integrated risk management	- Integrated risk management: organization-wide approach to managing risk at the strategic, operational, and project level - Apply sound risk management practices and fostering a working culture that values learning, collaboration, innovation, responsible risk-taking and continuous improvement	308	782
Act, Group, Law, etc.		235	464
What's being done (explanation or name)		183	207
The strategic risk communications process requires continuous improvement through evaluation	- Calls for clear, measurable objectives - Self-evaluated continuously to maintain excellence	18	20
